# Supplementary material for: The interactome of intact mitochondria by cross-linking mass spectrometry provides evidence for coexisting respiratory supercomplexes
Source: Mol Cell Proteomics. 2017 Dec 8;17(2):216–32. doi: 10.1074/mcp.RA117.000470 (PMC5795388; doi:10.1074/mcp.RA117.000470)
Supplement: Supplemental Data [file supp_RA117.000470_133922_0_supp_23978_fzffwf.zip › spectra_annotation/mito_spectra_annotation/7-1-2-1-1-1.pdf]

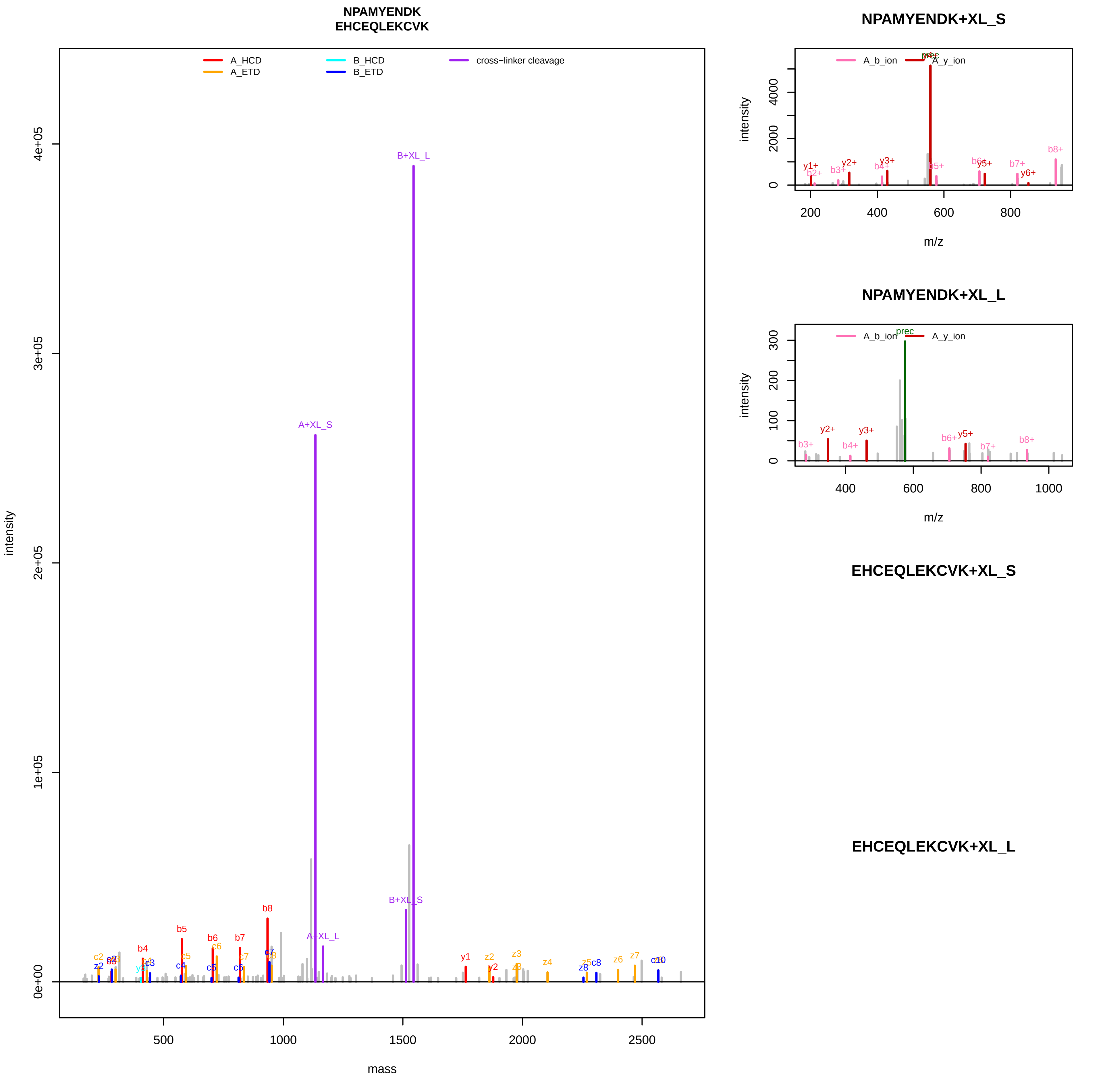
NPAMYENDK
EHCEQLEKCVK
A_HCD
A_ETD
B_HCD
B_ETD
cross−linker cleavage
B+XL_L
A+XL_S
B+XL_S
A+XL_L
b4
b5
b6
b7
b8
y1
y2
c2
c3
c4
c5
c6
c7
c8
c10
z2
z3
z4
z5
z6
z7
z8
intensity
mass
0e+00
1e+05
2e+05
3e+05
4e+05
500
1000
1500
2000
2500
NPAMYENDK+XL_S
A_b_ion
A_y_ion
prec
y1+
b2+
b3+
y2+
b4+
y3+
b5+
b6+
y5+
b7+
y6+
b8+
intensity
m/z
0
2000
4000
200
400
600
800
NPAMYENDK+XL_L
A_b_ion
A_y_ion
prec
b3+
y2+
b4+
y3+
b6+
y5+
b7+
b8+
intensity
m/z
0
100
200
300
400
600
800
1000
EHCEQLEKCVK+XL_S
EHCEQLEKCVK+XL_L
